# Microstructural and Enzymatic Contributions to Texture in High Pressure Processed Fruits and Vegetables

**DOI:** 10.3390/foods14183267

**Published:** 2025-09-20

**Authors:** Danielle Heaney, Olga I. Padilla-Zakour

**Affiliations:** Department of Food Science, Cornell University, Geneva, NY 14456, USA; dnh45@cornell.edu

**Keywords:** high pressure processing, fruits, vegetables, texture, pectin methylesterase, polygalacturonase, microstructure

## Abstract

High pressure processing (HPP) is common for beverage treatment, but its application to whole fruits and vegetables is more complex given their susceptibility to tissue softening and noticeable texture changes. Impacts of HPP highly depend on the food material, amount of pressure applied, length of exposure, and synergetic effects with temperature. This paper addresses the effects of HPP parameters (pressure, holding time, and temperature) on physical and chemical attributes, which are responsible for texture in non-beverage fruit and vegetable materials. Nonenzymatic attributes addressed include microstructure and quantitatively measured texture attributes (hardness, displacement distance, springiness, chewiness, cohesiveness, and resilience). Enzymatic attributes addressed include measures of pectin methylesterase activity and polygalacturonase activity, specifically changes to pectin composition and degree of esterification. Other parameters explored include recovery of texture during shelf life, HPP-assisted infusion with calcium and pectin methylesterase for improved texture, and the role of isoenzyme and matrix environment on texture. Based on findings in literature, HPP or combined HPP and thermal treatments has the potential to expand beyond the beverage sector into whole fruit and vegetable products for maintained or improved texture.

## 1. Introduction

Food processing methods are constantly evolving to meet consumer demands while ensuring the safety and quality of the product. Thermal processing is the standard method for plant-based fruit and vegetable products, controlling unwanted microbes and quality changes. Blanching and pasteurization, for example, are popular thermal treatments for commercial fruit and vegetable products like juices, purees, and jams. However, compared to the unprocessed product, heat treatments can cause detrimental quality changes like texture loss and degradation of vitamins and minerals, which [1], from a consumer perspective, are undesirable. Driven by consumer health initiatives and global sustainability, current trends indicate consumers desire minimally processed products with an emphasis on plant-based and clean-label ingredients [2]. Given this growth in consumer concern for a healthy diet via consumption of minimally processed foods and the quality changes associated with thermal treatments, alternative, nonthermal processing methods are increasingly being explored.

One such nonthermal method for producing safe foods with extended shelf life and enhanced nutritional quality is high pressure processing (HPP). During HPP, pressure up to 1500 MPa is transmitted uniformly to a pre-packaged product via a water source that fills the pressure chamber [3]. Under commercial conditions ranging from 400 to 600 MPa, this renders the food as safe to eat, as it leads to pressurized pasteurization that destroys vegetative pathogens, yeasts, and molds [3,4]. From a quality perspective, important processing parameters to consider when applying HPP include pressure, time (pressure buildup time, holding time, and pressure release time), temperature, and adiabatic heating. Though HPP is categorized as a nonthermal technology, it is still associated with about a 2–3 °C temperature increase per 100 MPa [3]. However, thermal degradation associated with HPP is much lower than that of heat treatments like pasteurization, given its overall lower operating temperature.

Relative to thermal treatments, HPP offers a mechanism to better preserve organoleptic properties inherent to raw fruits and vegetables. HPP extends shelf life and improves nutrition and sensory attributes, owing to its minimal effects on covalent bonds in low-molecular-mass flavors and colors [3,5]. HPP is also considered advantageous over thermal processing because it reduces off-flavors and Maillard browning. As a result, it can produce products with better sensory attributes than thermal treatments [6].

Industrially known plant-based products produced using HPP include blended or pureed juices, dips, sauces, and spreads, given their low pH and high water activity protect them against spore-forming pathogens in anaerobic packaging conditions [3,4]. However, to stay relevant and meet consumer demands, HPP needs to expand into new food categories. Specifically, exploring the application of HPP to whole fruit and vegetable (WFV) products aligns with the growth of the plant-based market space. Since maintaining texture is a key element in preserving WFV, addressing the effects of HPP on the enzymatic and nonenzymatic determinants of texture in WFV foods will make this technology more accessible to the food industry and provide insight into optimization techniques. Nonenzymatic determinants of texture include turgor pressure, membrane integrity, cell-to-cell adhesion, β-elimination reactions, and acid hydrolysis, while enzymatic textural changes are induced by pectinesterases like pectin methylesterase (PME) and polygalacturonase (PG), cellulases, peroxidases, and β-galactosidase [7,8,9].

Review papers that evaluate the role of HPP on texture, on enzymes, and on fruit and vegetable products exist [9,10,11], but no review to our knowledge evaluates the intersection of these variables within non-beverage applications. This review addresses how HPP parameters (pressure, holding time, and temperature) affect both enzymatic and nonenzymatic determinants of texture, specifically microstructure, pectin composition, degree of esterification, and measures of PME and PG activity. Evaluating the effects of HPP on these specific properties will help inform new applications of HPP in product development.

### 1.1. Physicochemical Aspects of Plant Cells That Influence Texture

#### 1.1.1. Cell Wall Structure

Textural properties in fruit and vegetable tissues are governed by the structure of the plant cell wall, and understanding this structure helps elucidate texture changes that take place during HPP. Plant cell walls are the protective components of plant cells that control the movement of solutes and solvents in and out of the cell. They consist of three main layers: the primary cell wall, the secondary cell wall, and the middle lamella. The primary cell wall is the innermost layer built from a cellulose-hemicellulose network containing pectin, bound enzymes, structural glycoproteins, phenolic esters, and minerals [8,12]. The middle lamella is the outermost layer between two adjacent primary cell walls. It contains pectin to improve firmness and elasticity and holds adjacent cell walls together. Together, the primary cell wall and middle lamella are responsible for the texture and structure of fruits and vegetables. Turgor pressure from water within the cell also plays a role.

Pectin is made up of polymeric galacturonic acid residues linked via α-D-(1-4) linkages, with some branches on its backbone made from neutral sugars like D-xylose, L-rhamnose, L-arabinose, and D-galactose [13]. These galacturonic acids are esterified, and the degree of methylesterification and branching of neutral sugar side chains varies among different types of pectin [13]. Analysis of the cell wall involves its division into cellulose, hemicellulose, and pectin components [7]. Characterization of its pectin composition involves further division into different fractions, including water-soluble pectin (WSP), chelate-soluble pectin (CSP), and sodium-carbonate-soluble pectin (NSP). WSPs are polymers dissolved in the apoplast that are loosely bound to the cell wall with ionic and non-covalent bonds [7,14]; CSPs include calcium-pectates, which are also ionically integrated into the cell wall matrix through cross-linking, typically in the middle lamella [7,14,15]; NSPs contain covalently bound pectins, particularly neutral sugars like rhamnogalactorunan I from the primary cell wall [14,15]. The amounts/ratios of these fractions vary with ripening and resultingly impact firmness [15].

#### 1.1.2. Pectin Methylesterase and Polygalacturonase-Induced Changes

**Figure 1:**
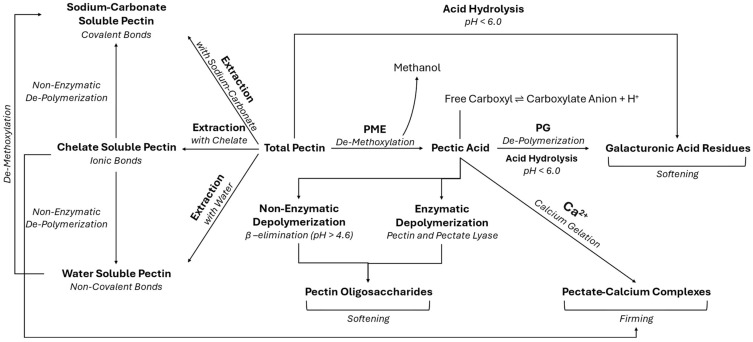
Mechanisms in the cell wall that govern enzymatic and non-enzymatic changes to texture in pectin-based foods.

PME and PG are responsible for changes in pectin, which lead to texture softening during ripening or food processing. Figure 1 depicts a simplified version of the potential pathways of PME and PG in the cell, different mechanisms of change to pectins, and their effects on texture. Together these enzymes create hydrolytic changes in pectin fibers, allowing for tissue softening as the cell wall weakens and the middle lamella polysaccharides solubilize. During this process, PME de-esterifies pectin into pectic acid, creating methanol and free carboxyl groups; PG then depolymerizes this pectic acid into galacturonic acid residues by cleaving the α-(1-4) glycosidic bonds [9]. This results in tissue softening of the plant material as structural integrity is lost. Thus, PME and PG together have a softening effect on tissues.

To prevent such softening, which is typically undesirable during food storage, reducing hydrolysis of pectic acids is key. This is achieved through inactivation of PG to minimize enzymatic hydrolysis using methods like low-temperature blanching. PG is thermolabile at temperatures above 55 °C [6], meaning low-temperature blanching inactivates PG. However, pH and temperature conditions need to be further adjusted to control non-enzymatic hydrolysis. Under acidic conditions (pH < 6.0 for pectate and pH < 4.5 for pectin), softening due to acid hydrolysis can still occur [16,17]. Under alkaline pH (>pH 4.5) and elevated temperatures (>80 °C), β-elimination reactions can cause unwanted softening [5,18,19]. β-elimination reactions involve the non-enzymatic depolymerization of pectin. Substituents are lost from two adjacent carbon, nitrogen, or oxygen atoms on pectin, and an unsaturated bond reforms in their place [5]. β-elimination reactions may be reduced directly through temperature and pH control or indirectly through enhanced PME activity. Sensitivity to thermal depolymerization via β-eliminations decreases at lower degrees of pectin methoxylation, meaning demethoxylation by PME may assist in inhibiting β-eliminations [20]. Acid hydrolysis tends to occur more rapidly under acidic conditions than β-elimination reactions, and pectate tends to be more rapidly susceptible [16,17]. On the other hand, because pectate lacks esterified methyl ester groups, it is not susceptible to β-elimination reactions [16,17]. Pectin and pectate lyases are enzymes that can also cause cleavage of pectin through a β-elimination pathway [21].

Another tactic for preventing softening during food processing is through the use of pectinases as processing aids. By adding PME to canned fruits and vegetables, texture can be improved through the calcium-dependent gelation of pectin via a concept known as the egg-box model. During this process, two antiparallel pectin chains form dimers with calcium and aggregate. In juices and fruit- or vegetable-based beverages, this binding of calcium to de-esterified pectin translates to a negative effect. Juice separation is apparent as the juice becomes clear and aggregated pectin fibers settle, or precipitate, to the bottom of the juice. However, in WFV, in which a firmer product is desired, this gelation or cross-linking has a positive effect and enhances firmness. Ultimately, controlling PME activity while eliminating PG activity and β-elimination reactions is the favorable pathway for enhanced texture of whole fruit and vegetable products.

## 2. Enzymatic Changes Induced by High Pressure

### 2.1. Effects of High-Pressure Processing Parameters on Enzyme Activity

To better understand how HPP influences food texture, this section examines the effects of pressure, holding time, and temperature on the activity of two key cell wall-modifying enzymes: polygalacturonase and pectin methylesterase.

#### 2.1.1. Pressure and Holding Time: Polygalacturonase

PG is pressure sensitive and can be completely inactivated by increasing pressure and holding time. For instance, two purified PG isoforms from four tomato varieties were pressure sensitive and inactivated beginning at 300 MPa for 15 min and completely inactivated at 500 MPa for 15 min [6]. Likewise, Tangwongchai et al. noted inactivation of PG around 500 MPa [22]. However, in persimmons, PG activity varied by holding time, pressure, and persimmon astringency [23]. PG activity in astringent persimmons was maintained at 200 MPa, while all high-pressure treatments (200–400 MPa, 1–6 min, 25 °C) lowered PG activity in non-astringent persimmons [23]. The difference in conditions leading to inactivation in each of these WFV formats emphasizes the importance of food matrix composition in enzyme activity, discussed further in Section 5.

#### 2.1.2. Pressure and Holding Time: Pectin Methylesterase

On the other hand, low to intermediate pressures tend to enhance or maintain PME activity, and survival at higher pressures indicates baroresistance. Pressures from 100 to 400 MPa maintained or enhanced measures of PME activity relative to untreated WFV, particularly between 20 and 55 °C, as seen in carrots and astringent and non-astringent persimmons [23,24]. As pressures approach and exceed 400 MPa, PME can stay active, though activity may decrease. Trejo Araya et al. demonstrated this, such that increasing pressure enhanced carrot PME activity compared to raw values, but once pressurized to 400 MPa and above, activity decreased [25]. These effects appear to be dependent on the WFV matrix, with purification of PME leading to even greater baroresitance. Purified carrot PME remained pressure resistant up to 600 MPa for 15 min at and below 40 °C [24]. PME from carrot pieces required 800 MPa, 40 °C, and 75 min for a one-log reduction in activity [26]. Crude tomato PME at 25 °C was also extremely pressure resistant, only beginning to inactivate at 700 MPa for 15 min and retaining 50% activity at 850 MPa for 15 min [6].

Holding time is positively correlated with PME activity, but there may be a threshold above which it is too high. Higher holding time (5 min) tended to have greater PME activity in apples [27], but increasing holding time above 10 min in carrots tended to have decreasing effects [25]. Treating zucchini at 400 MPa for 5 min yielded the highest residual PME activity (86 ± 7.5%), and increasing holding time from 1 to 5 min increased activity [28]. However, increasing holding time at higher pressure (600 MPa) decreased PME activity [28], suggesting PME activity depends on both holding time and applied pressure.

Ultimately, PME appears to be more pressure resistant than PG, and pressure and holding time have varying effects. PME activity was generally enhanced with increased pressure conditions at optimal temperature, though above 400 MPa activity began decreasing. PG was generally inactivated above 300 MPa and more sensitive. There are significantly fewer published studies on the effects of HPP on PG, potentially due to its ease of pressure inactivation. More studies are needed to realize the full effects of PG in different matrices.

### 2.2. Synergetic Effects of High-Pressure Processing with Temperature

In addition to pressure and holding time, synergetic effects of pressure and temperature affect enzyme activity. When combining pressure and thermal treatments, studies found that PME and PG have an optimal operation temperature for maximum activity. Optimum temperature and pressure may enhance enzyme activity more than at other temperatures. Authors describe this as an “antagonistic” effect of pressure and temperature on inactivating PME [24,29]. In other words, pressure and temperature have a negative effect on PME inactivation.

Verlent et al. studied the effects of temperature (30 °C to 70 °C) and pressure (100 MPa to 500 MPa) on tomato PG and PME extracts [30]. For pressures up to 300 MPa, increasing temperature increased both PG and PME activity [30]. Pressures above 300 MPa tended to decrease activity, and temperatures above optimum also decreased activity [30]. For PG, the optimum was determined at 50 °C; for PME, the optimum was determined at 60 °C [30].

Similar results were found in carrots, in which PME activity increased with optimum pressure and temperature conditions [24]. In shredded carrots, PME activity was most active at 50 °C between 200 and 400 MPa, while whole carrots’ PME activity was optimum at 60 °C between 100 and 400 MPa [24]. This likely relates to the conformational structure of carrot PME and PME’s tendency to inactivate at 80 °C [31]. Some authors describe that with increasing pressures, the denaturation temperature of proteins decreases, and vice versa [32]. This could explain why PME and PG were heat sensitive at lower temperatures as pressure increased. Other authors suggest PME may be more active under combined optimal pressure and temperature conditions due to its ability to undergo reversible conformation changes or due to the extent to which heat penetrates the matrix tissue and ultimately acts upon the enzyme. Enzyme activity highly depends on enzyme conformation and affinity for substrate. Under pressure, conformational volume changes favor taking up less space. Thermal processes also lead to protein denaturation and potentially aggregation as it breaks down covalent bonds [33]. However, when used simultaneously, Ly-Nguyen et al. describe that low pressure (≤300 MPa) and mild/high (>50 °C) temperatures may have complementary effects that stabilize enzymes against inactivation [29]. Namely, HPP and thermal treatments oppositely alter the interactions between enzymes and water molecules. Whereas heat causes the loss of water molecules during thermal processing, high pressure hydrates the charged and nonpolar groups of amino acid residues exposed on unfolded proteins [29]. Authors refer to these opposing reactions as having a stabilizing effect on enzymes [29]. Thus, high pressure may stabilize against water losses as pressure rises.

Clearly, there is a temperature-pressure dependency for enzyme activity, such that low pressures may enhance enzyme activity near optimal temperature but inactivate enzymes under high-temperature treatments.

### 2.3. Relationship Between Enzymatic Activity and Texture

The past few sections described the individual effects of pressure PME and PG activity. However, fewer studies directly evaluate the relationship among these three variables under pressure [25,27,34,35,36,37,38,39], indicating an area for improvement. In studies that evaluated these effects, decreased PG activity was typically associated with firmer texture, though the effects of PME activity varied. Compared to untreated controls, high-pressure-processed (HPPed) samples’ residual PME activity was either enhanced, maintained, or only slightly lowered [23,24,25]. Despite similarity in PME activity, HPPed samples were not necessarily as firm. Even when HPPed samples had comparable PME activity to controls, there was still an initial firmness loss in some cases, confirming the role of the initial pulse pressure in turgor loss regardless of PME activity [25]. PME activity above that of untreated samples also did not necessarily increase firmness, and PME activity below that of untreated samples did not necessarily indicate firmness loss. For example, in HPPed green peppers, an increase in PME activity compared to untreated peppers was not associated with increased firmness, and a significant reduction in PME activity from blanching was not associated with significantly decreased flesh firmness [35]. It is possible that the untreated matrix serves as a texture threshold for maximum firmness, and initiation of other factors like β-elimination reactions, rather than loss in PME activity, correlates more strongly with textural changes. One texture parameter, displacement distance (rubberiness), was negatively associated with PME activity [28]. These findings suggest other factors interplay complexly with PME activity in ways that are not fully predictable. More research is needed evaluating the combined effects of pressure, PME, and PG on texture to delineate these interactions.

### 2.4. Changes to Pectin Composition and Degree of Methylesterification After High Pressure Processing

HPP leads to disruption of the cell membrane, which could improve interactions between substrate and enzyme and alter pectin composition. Therefore, measurements of pectin fractions and degree of methylesterification (DM) before and after HPP shed additional light on the changes to WFV texture after HPP. A decrease in DM can improve texture by allowing for calcium bridging and indirectly suggests higher PME activity. Temperature and pressure combinations can also influence DM and reduce β-elimination reactions, improving PME activity and texture. De Roeck et al. and Sila et al. found treatment with both pressure and temperature reduced the DM in carrots [36,40]. Treatment at 200 to 500 MPa at 60 °C for 15 min yielded a lower DM with higher textural hardness [36]. Because the DM was low, this also prevented opportunities for β-elimination reactions despite the higher temperatures, ultimately improving the hardness of the carrot samples [1]. The same was found in carrots pre-treated with HPP (400 MPa, 15 min, 60 °C) prior to thermal processing (110 °C) [37]. In other words, pectin thermostability is influenced by HPP.

Though lower DM is generally associated with increased PME activity and firmness, this is not always the case. In carrots, DM decreased in HPPed samples (200–550 MPa, 2–30 min) compared to untreated samples due to a pressure-dependent galacturonic acid extraction, and residual PME activity was maintained or increased (100–300 MPa, 2–10 min) [25]. However, these effects did not correlate with significantly increased hardness; instead, sample softening was attributed to tissue damage [25]. Others similarly found no apparent relationship between DM, pressure, and hardness [36,41], except in combination with temperature, during which 60 °C saw decreasing DM with increasing pressure [36]. This lack of correlation between DM and texture in some WFV suggests the tissue softening effect from the initial pulse pressure may take precedence over biochemical responses when it comes to mechanisms responsible for texture changes. For example, demethylated pectin has been shown to have limited binding abilities with endogenous cell wall-bound calcium [42]. Calcium is essential for gel formation in de-esterified low methoxy pectin [43], but there may not be enough endogenous calcium or salts to encourage bridging in some systems regardless of PME activity and DM. On the other hand, in kohlrabi pickled with added divalent salt ions to assist with bridging, HPP decreased the DM compared to untreated and thermal controls and improved texture compared to the thermal control [44]. HPP has also been shown to have quick de-esterifying abilities and create strong Ca^2+^ gels from low methoxy pectins [43].

In addition to DM, measuring cell wall polysaccharide composition through total pectin, WSP, CSP, and NSP provides meaningful data. The presence or absence of pectin in samples impacts their post-processing firming. A lack of pectin, as seen in celery, led to texture softening after HPP regardless of calcium content [45]. However, in samples with pectin, HPP caused less damage to the linear pectin chain than thermal treatments, which broke it into shorter chains and led to higher pectin content [44]. These effects correlated with improved texture in HPP samples [44]. The content of the total pectin also matters. Several studies show a higher CSP content and lower WSP content in HPP samples compared to thermally processed samples immediately after processing [40,44,46]. This may result from depolymerization of CSP into WSP and an increased ionic cross-linking from reduced DM [40,44,46]. Such a process weakened the texture of thermal samples, while HPP samples contained higher CSP and had better texture [44,46]. This is because CSP contains stronger ionic bonds, unlike WSP, and contributes to strengthening the cell adhesion in the middle lamella [15,44]. One divergent study found that when HPP (400 MPa, 15 min, 60 °C) was used as a pretreatment before thermal processing (90 °C, 4 min), WSP decreased and NSP increased, while there were no changes in CSP [18]. Changes to NSP are mixed. Several studies found the NSP content to be greater or equal in HPPed samples [18,40,44], but one found thermally processed samples to have higher NSP content [46]. Changes in pectin fractions have been shown to impact texture, likely due to non-enzymatic depolymerization (β-eliminations or acid hydrolysis) or demethoxylation, which influences the chemical bonds between pectin and the cell wall, reducing pectin solubility in water and allowing for conversion to different fractions [18,46]. WSP fractions have been shown to be more susceptible to β-eliminations [18]. These findings suggest the matrix composition before HPP plays an important role. There is a need for more research into the relationship between DM and changes to turgor pressure after the initial HPP pulse to understand the precedent mechanism (biochemical or physical) behind textural changes.

## 3. Nonenzymatic Changes Induced by High Pressure

### 3.1. Microstructure

Pressure treating WFV samples leads to changes in cell structure compared to untreated tissues, which can lead to textural changes. As pressure is applied, cells are compacted and intercellular gas is pushed out, leading to a reduction of intercellular spaces [47,48]. Cell membranes are deformed or broken by pressure treatments, and turgor pressure is lost, leading to solubilization of cell components through intercellular leakage [25,47]. Loss of turgor pressure is measured visually with scanning electron micrographs, transmission electron micrographs, and optical microscopy [22,28,44], and quantitatively through measurement of water loss of tissues after processing. Table A1 summarizes findings for studies evaluating the microstructure and/or texture of fruits and vegetables after HPP. In these studies, HPP caused changes in cell membrane integrity [25,48], cell elongation [25,49], and membrane permeability [25,50,51]. Water loss and/or ion leakage due to membrane rupture after HPP was seen in pineapple, onions, and pickled kohlrabi [44,51,52]. Increased water loss, or juiciness, cell wall swelling, and loss of cellular adhesion are also signs of softening during ripening [15].

Compared to cell membranes, cell walls generally remained intact but deformed after HPP. Pressure treatments (≥100 MPa) have led to deformation, thickening, and dehydration of cell walls [34,49]. Evidence of increased middle lamella dissolution and cell-cell debonding was seen in pumpkins (300 and 600 MPa, 2 min) and carrots (300 MPa, 2 min and 550 MPa, 30 min) [25,41]. In peaches (600 MPa, 5 min) and carrots (100–550 MPa, 2–30 min), cell wall deformation such as swelling caused by increased cell permeability occurred, but not cell wall breakage [25,47]. In some matrices, this deformation increased with increased pressure [25,41]. Cell wall changes were pronounced when increasing holding time to five or more minutes [25,28], and cell wall breakdown was only seen in pressure treatments (550 MPa) above five minutes [53]. These findings suggest holding time may play a more significant role in cell wall breakdown.

Despite these changes in microstructure to the cell membrane and walls, HPPed samples experience less damage than thermally treated fruits and vegetables. HPPed samples (600 MPa for 3 min in pineapples and 400 MPa for 20–30 min in pickled kohlrabi) had smaller intercellular spaces and less cell shape irregularity compared to thermal treatments (93.3 °C for 5 min in pineapples and 90 °C for 30 min in pickled kohlrabi) [34,44], highlighting that HPP is still a useful alternative to thermal treatments.

Some hypothesize the microstructure changes caused by HPP could be due to the initial pulse pressure when HPP is applied [25,50,54,55]. This initial pressure application is believed to cause the loss of turgor pressure and intercellular leakage noted in several samples and is also associated with an “initial pulse softening.” Initial pulse softening is described as an instant loss in tissue firmness due to pressure application [25,51,55]. Some samples, like carrot, celery, green pepper, and red pepper, experienced a greater initial pulse softening with increased pressure (100–400 MPa) [55]. Other studies suggest there is a maximum pressure above which relatively few changes occur. In peaches, carrots, and tomatoes, HPP damaged cells and caused loss in turgor and firmness, but increasing pressure above 300 MPa did not exacerbate these softening effects [22,25,51]. Hernández-Carrión et al. also found fewer changes in microstructure once reaching 500 MPa in red sweet pepper [49]. One explanation as to why cellular changes do not occur at pressures above a certain level was proposed by Trejo Araya et al., regarding the physiochemical properties and compressibility of water [25]. Water can be compressed up to a certain percentage of its volume—up to 4% at 100 MPa and 15% at 600 MPa at room temperature [56]. Foods with high water content follow compressibility patterns close to water [56]. This suggests that increasing processing pressure does not increase the water compressibility in cell membranes, and thus, the texture and extent to which cell membranes are disrupted under increasing pressure remain the same.

Overall, HPP causes an initial loss in firmness in WFV, but this loss may not be exacerbated at higher pressures and mostly affects the cell membrane, not the cell wall, where structural and textural integrities derive.

### 3.2. Texture

#### 3.2.1. Hardness

To understand the effects of the initial HPP pulse and enzymatic effects on softening, texture can be quantitatively measured. One significant texture parameter measured for fruits and vegetables is the peak force, or hardness, measured through compression, puncture, or cut tests. Hardness is often synonymous with firmness and indicates the crispness or ease of biting through a sample, or resistance to deformation. Per Table A1, most HPPed samples were significantly softer than untreated samples, likely due to the loss in turgor pressure and initial pulse softening. In sweet potato, cocoyam, carrot, sweet green bell pepper flesh, zucchini, pumpkin, and pineapple, the maximum force decreased for HPPed (100–600 MPa) samples compared to raw samples [25,28,34,35,41,57].

Compared to thermally treated samples, HPPed samples tend to be insignificantly different or firmer. During a puncture test, the peak force of all HPPed (400 and 600 MPa, 1 and 5 min) zucchini samples was insignificantly different from blanched (90 ± 2 °C, 2 min) except for one (400 MPa for 1 min), indicating that HPP provides similar texture to blanched samples under certain conditions [28]. HPP sweet green bell pepper also had no difference in skin firmness compared to thermal treatments, except the most intense condition (98 °C, 2.5 min), which was softer [35]. Other studies found that HPPed WFV performed better than thermally treated ones in terms of hardness. In carrots, HPP (600 MPa, 2 min) corresponded to a 44% loss in hardness during a compression test relative to raw carrots, whereas thermal processing (100 °C, 20 min) led to a 96% loss in hardness; the high-pressure-treated carrots also had a slightly higher peak cutting force than raw carrots [54]. In a compression test of peaches, all high-pressure treatments (600 MPa, 5, 10, and 30 min) had greater hardness than thermal treatments, and increasing HPP holding time enhanced hardness [46]. HPP green beans were also firmer than canned green beans in a cut test [58]. Improvement of hardness in HPPed samples compared to thermal samples is more likely caused by a decrease in β-elimination reactions at colder processing temperatures, a compacting of cells under high pressure, and potentially the activation of pectinases.

Some studies found divergent hardness trends, like in red Lamuyo-sweet peppers and persimmons [49,59]. In these cases, HPP (100–500 MPa for 15 min in sweet peppers and 200 MPa for 6 min in persimmons) reduced the hardness compared to thermally treated controls (70 °C for 10 or 15 min) [49,59]. This could be due to a lower temperature condition (70 °C) used for the thermal treatment compared to other studies, which does not encourage β-elimination reactions. For the red Lamuyo-sweet peppers, this could also be because the pepper variety was inherently low in PME activity and high in PG activity [49]. Even if the high pressure inactivated PG, there would be little firming effect from pectin bridging because there was no PME to first facilitate the pectin demethylation.

#### 3.2.2. Displacement Distance

In addition to hardness, the displacement distance at peak force also tends to increase under HPP, meaning the texture analysis probe travels more distance before the sample breaks. While a longer displacement distance typically is a sign of increased or non-uniform hardness, it can also be a sign of increased deformability through resistance to breakage. In carrots and peaches, the displacement distance measured through a cut test increased for HPPed samples compared to raw samples [25,53]. In all cases, a shift in displacement distance was associated with a rubbery sample, which could be because the instantaneous pulse softening during HPP caused a loss of turgor pressure, altering membrane permeability and water composition, giving the cells a “soaked,” wet, or rubbery-like appearance or texture [25,48]. Some authors hypothesize the soaked appearance of HPP WFV results from water movement through the cell wall after turgidity loss [53]. This can be verified by other studies, which found that HPP increased the juiciness [54] and ion leakage [52] compared to untreated samples, but not moisture content [28], indicating the rubberiness derives from a change in water and ion distribution.

The abovementioned studies compare displacement distances of HPPed samples to those of untreated samples, but comparison between high-pressure and thermally treated samples is key to understanding whether this rubberiness is an undesirable attribute or insignificantly different from the texture produced by traditional thermal processing methods. For example, Paciulli et al. found that HPP (400 and 600 MPa, 1 and 5 min) significantly increased the displacement distance compared to the untreated samples, but the displacement distances for the HPP and thermally treated (90 °C, 2 min) samples were insignificantly different [28]. This suggests that increased rubberiness is not abnormal during processing and may not be undesirable in HPPed samples. In fact, sensory panels have interpreted this increase in rubberiness as a flexibility parameter in HPPed carrots (600 MPa, 2 min) and found them to be equally as crunchy as sous vide carrots (90–95 °C, 5 min) even though they had a slightly greater displacement distance [54].

#### 3.2.3. Springiness, Chewiness, Cohesiveness, and Resilience

HPP has mixed effects on other texture profile analysis parameters, including springiness (the ability of the product to reshape after deformation), cohesiveness (the force required to break the product into separate parts), chewiness (a representation of resistance during chewing), and resilience (the ability of the product to return to its original shape). HPP ranging from 100 to 600 MPa and 2 to 30 min led to insignificant differences or increased springiness compared to untreated and thermal controls (90–100 °C, 2–30 min) [34,52,54,59]. Chewiness (400 and 600 MPa, 3–30 min) was the same or decreased compared to untreated controls; cohesiveness and resilience (600 MPa, 3 min) were the same as untreated controls; and chewiness, cohesiveness, and resilience (100–600 MPa, 2–30 min) were the same or increased compared to thermal controls [34,52,54].

Ultimately, while turgor pressure and β-elimination reactions play an important role in the increased hardness and displacement force, the varied effects of HPP on springiness, chewiness, cohesiveness, and resilience highlight the nuances in texture depending on the matrix compositions.

### 3.3. Recovery and Shelf Life

Some fruit and vegetable samples can recover lost hardness following the initial pulse pressure during holding time and shelf life [25]. Although cell structure changes occurred, recovery of texture through tissue firming at low-pressure treatments was seen in carrots, peppers, celery, pears, pineapples, and oranges [55].

High-pressure-treated WFV can also maintain textural qualities during shelf life. Hardness of HPPed green beans and hardness, cohesiveness, and chewiness of peaches were retained better than in untreated samples over 31 days and 21 days in cold storage (10 °C), respectively [58,60]. The authors suggested this slower loss in textural properties over time could be the result of PG inactivation under pressure [60]. Other studies on peaches also found that HPP-treated samples maintained better hardness over 180 days than thermally treated samples, particularly when kept in cold storage (4 °C) relative to 25 °C [46]. Zhang et al. mention that storage at 4 °C helped slow down the depolymerization of intercellular pectin, which could cause cell wall loosening [46]. The peach samples that underwent longer holding times also had better hardness retention over shelf life [46]. Demethylation via PME also depends on storage time—in pickled cucumbers, firmness needed to stabilize over time, and PME demethylated until no further demethylation could be achieved [42].

## 4. Enzyme Infusion and Added Calcium

In systems with low endogenous PME and/or calcium, impregnation can improve textural conditions. Enzyme infusion is explored in WFV and has proven promising in lowering DM and increasing hardness [61]. HPPed strawberry firmness was comparable to untreated when infused with PME, and infusion significantly reduced DM and limited tissue disruption [61]. In addition, HPP improved the calcium infusion ability compared to standard methods by assisting in infusing three times the amount, and even more when combined with PME pre-treatment [62]. Calcium infusion alone has promising effects in increasing or maintaining hardness for some HPPed samples [62,63]. HPP-assisted calcium infusion correlated with changes in cell wall diameter and increased cell damage positively affected infusion ability as it allowed more calcium to permeate cells [45]. Some authors found that calcium soaking after HPP (rather than before) also reduced texture degradation, as disrupted cells could better uptake calcium [36]. Others found calcium to travel through transport tubes or diffuse into cells [45].

## 5. Isozyme Stability and Matrix Composition

Given the differing enzyme activation and firming effects under high pressure, it is clear that the matrix composition of each fruit and vegetable differs and plays a significant role in texture. For example, two seemingly similar fruits (sweet red and green bell peppers) can have vastly different endogenous PME activities [35]. Moreover, Perera et al. demonstrated the importance of composition rather than residual PME amount in apples, given that apples with higher residual PME activity did not necessarily have the highest firmness [27]. Environmental conditions within cells, like pH, ionic composition, and cell structure, all determine how well enzymes and substrates will work together, and properties of individual isoenzymes found in the WFV matrices play an essential role in their measured activities. Some authors hypothesize the reason PME and PG are activated or deactivated under different conditions in different matrices is because of the presence of multiple isoforms [64]. Different isoforms have different molecular weights and isoelectric points, which influence their pressure, pH, and heat stabilities. Katsaros et al. explain that there may be at least two types of PME present in fruits and vegetables—one that is heat tolerant and another that is heat labile [64]. Other authors observed that the heat-tolerant isoform may correspond to the pressure-resistant form of PME [65]. Ly-Nguyen et al. reported purified carrot PME is composed of 5% to 6% of a thermostable fraction [29]. PG also has a thermolabile (PG2) and thermostable (PG1) fraction, particularly in tomatoes, and their β-subunit dictates their stabilities [6].

The isoelectric point and optimal pH conditions for different isoforms are sensitive to external conditions. pH influences the efficacy of de-esterification in the system and controls ionization, ultimately influencing enzyme activity, substrate binding, and ion crosslinking. The isoelectric points of PGs tend to be high, and Duvetter et al. refer to it as a basic protein [66]. The isoelectric points of PMEs can range from 6.0 to above 11 [66]. Optimum pH for WFV PME was found to be 7.0–8.0 [24,31], with activity ranging from pH 4–9 [22,24,31]. Addition of other components to the matrix, like salts, can shift these optimum conditions. For example, increasing cation concentration increases PME activity, up to a certain limit [67]. Pectin gel strength after demethylation by PME is also affected by pH. In a model system, gel strength increased from pH 2.5 to 3.5, likely because increasing pH increased pectin’s charge density and ionic binding ability to calcium [43]. Strength decreased above 3.5, likely because too much ionization strained hydrogen bonds [43]. Implications of this information for product development are that creating a product below optimum enzyme pH will lead to low or no activity in that product [31]. This limits the opportunities to enzymatically modulate texture and may explain why some studies find enhanced PME activity with no change in firmness. Effects of pH need to be individualized for products.

In addition to isoenzyme and matrix composition, studies have proven that the purification of the enzyme and the matrix determines its sensitivity to HPP parameters. PME in situ in carrots was more pressure resistant than purified carrot PME and carrot juice PME, which authors attribute to stabilization through cell wall factors and binding of the enzyme to the cell wall [24,26]. Purified PME was also less barotolerant than PME in a tomato matrix [22]. Enzyme activity and stability under pressure differ in model systems versus real food matrices. The conditions within a plant cell are naturally and precisely balanced to protect the enzyme, making it an ideal system for using HPP to control texture, as opposed to shredded, pureed, or juiced products.

## 6. Research Needs and Improvement Opportunities

Many interconnected mechanisms play a role in texture changes to WFV during HPP (pectin fraction/solubilization, PME activity, PG activity, DM, turgor pressure loss/initial pulse softening). Beneficial future studies would evaluate these relationships in intact fruit or vegetable matrices to address the emerging trends in WFV snacks. Evaluations in acidified WFV would be especially beneficial, since research on this topic is limited. Controlling biochemical properties like PME and PG activity during pressurized pasteurization could improve quality and maintain safety and shelf life, particularly if PME and/or calcium infusion is used. However, manipulating texture through these enzymes appears to be less predictable and highly dependent on the matrix composition. As a result, a more systematic approach needs to be taken to study relationships among these mechanisms and understand which enzymatic and nonenzymatic reactions take precedence in different matrices. Manipulating turgidity and protecting microstructure through methods like adding water-retaining agents to control water loss [44], controlling compression, decompression, and pressure cycling, and optimizing calcium and enzyme infusion could be potential options for creating novel textures in plant-based WFV products processed using HPP.

One downside of HPP is its cost. HPP is more expensive than traditional thermal processing. This is largely due to its high operational electricity requirements and high capital cost [68]. HPP is not always feasible from an industrial point of view. Given this, use of HPP to inactivate PG to prevent degradation of pectin and softening may not be as economically practical as thermal methods. Since PG is both thermally and pressure sensitive, thermal treatment may be more realistic. However, when it comes to enhancing or maintaining PME activity to encourage pectin gelation and firming effects, HPP may still be a better option. While low-temperature blanching is a common method for activating PME, it can lead to color degradation or changes in nutrition [68,69]. Since HPP generally has better quality preservation effects, it may mitigate some of these issues. Directly studying both production costs and how the improvements in product quality from HPP dictate product cost would be useful in clarifying the practicality of this method. Ultimately, this decision is a tradeoff of quality attributes, consumer desires, and cost, to name a few.

A second consideration for the practicality of this technology on WFV products is food safety. Generally, greater than or equal to 400 MPa for 5 to 10 min in cold conditions (under 20 °C) may be required in order to ensure pressurized pasteurization of a fruit product [70]. However, as seen in studies cited in this review paper, PME is sensitive to inactivation near these pressures in some products. In these cases, using HPP as a pretreatment for quality, in combination with hurdle technology for microbial safety, is an option. However, hurdle technology opens opportunities for thermal degradation, which negates some of the benefits of using HPP to begin with. Thus, this process would be best designed for high-acid foods, like whole fruit blends for yogurts, fruit cups, acidified salsas, or pickled vegetables. This poses a solution since the high acidity helps with microbial safety, while the HPP treatment can be used for quality enhancement.

## 7. Conclusions

In conclusion, HPP holds potential to be a useful tool for regulating the texture of WFV products, though the right conditions highly depend on the matrix type, ratio of thermostable to thermolabile and pressure-stable to pressure-labile enzyme fractions, and synergy with temperature. Texture changes are mainly due to turgor loss and a reduction in thermal softening from β-elimination reactions. Enzymes PME and PG also play a role in texture, as measured through residual activity, DM, and pectin fractions, but their effects are more complex and depend highly on fruit and vegetable variety. PME was generally activated with increasing pressure up to about 400 MPa, at which point some deactivation began. PG was generally much more pressure sensitive, beginning to deactivate closer to 300 MPa. Pressure also lowered the thermal inactivation temperature, and inactivation occurred once exceeding the optimum temperature for enzyme activity. Overall, the hardness of HPPed samples was the same as or improved from that of thermal samples but less than that of raw samples due to disruption of cell membranes by an initial pulse pressure. Displacement force distance was increased, indicating HPP samples were more rubbery than untreated samples, but not necessarily undesirable. However, the observation that texture softening occurred in low-pectin celery regardless of calcium content highlights a key limitation of HPP-assisted firming: its effectiveness is constrained in matrices lacking sufficient pectin for structural reinforcement. Future work could benefit from studying the role of HPP-assisted firming in more matrices with high and low pectin and PME content. Useful research would include more direct evaluations of the association between pectinase activity and texture in HPPed WFV.

## Data Availability

The original contributions presented in the study are included in the article, further inquiries can be directed to the corresponding author.

## Appendix A

**Table A1:** Effects of high pressure processing (HPP) on texture and microstructure of whole fruit and vegetables.

Matrix	HPP Conditions	Control Conditions	Texture		Microstructure		
			Effects Compared to Control(s)	Analysis Method(s)	Effects Compared to Control(s)	Analysis Method(s)	Reference
Apple	100 and 200 MPa, 15–60 min	Untreated	Initial pulse softening increased with pressure; Increased holding time increased firmness at 100 MPa	Single compression	n.d.	n.d.	[55]
	600 MPa, 1–5 min, 18–22 °C		Granny smith apple: Maximum force increased with holding time; Pink lady apple: no difference	Puncture	n.d.	n.d.	[27]
Asparagus	10–600 MPa, 0.5–30 min, 20–38 °C	Untreated	Slight (≤100 MPa) to significant firmness loss (400–600 MPa); Increased holding time led to recovery	Puncture	Altered cell shapes and disorganization; 400 and 600 MPa showed signs of cell rupture	Scanning Electron Microscope (SEM)	[38]
Blueberry	200 and 600 MPa, 5–60 min, 3 °C	Untreated	Hardness decreased, except at 600 MPa, 60 min which was the same; 600 MPa treated samples were firmer than control after one week	Double compression	n.d.	n.d.	[71]
Carrot	100–400 MPa, 5–60 min	Untreated	Initial pulse softening increased with pressure; Increased holding time increased firmness (100 MPa)	Single compression	n.d.	n.d.	[55]
	Pre-treatment: 0.1–500 MPa, 15 min, 20–60 °C Treatment: 90–110 °C, 0–80 min	90–110 °C	Texture degradation rate decreased with increased pre-treatment temperature and pressure; Increased processing time led to the same final hardness; Calcium soaking increased hardness	Single compression	n.d.	n.d.	[36]
	100–550 MPa, 2–30 min, ≤39 °C	Untreated	Hardness decreased, with no further losses > 300 MPa; Longer holding time (300–500 MPa, 30 min) increased hardness; Displacement distance and force to cut increased	Single compression; Cut	Cell to cell contact decreased and cell wall deformation, buckling, folding, and elongation increased with pressure	Light Microscope (LM)	[25]
	Pre-treatment: 400 MPa, 60 °C, 15 min Treatment: 90–110 °C, ~0–140 min	90–110 °C, ~0–120 min	Thermo-softening rate constants decreased; Higher residual hardness values	Single compression; Cut	Less cell-wall swelling	LM	[37]
	600 MPa, 2 min	Untreated	Hardness decreased, except day 14 which was comparable to control; Peak cutting force was slightly higher; Displacement distance was greater	Single compression; Cut; Three-point bend	Less compact and less organized cells	Cryo-SEM	[54]
		Sous-vide, 90–95 °C, 5 min	Hardness decreased and less recovery over 14 days; Peak cutting force was significantly higher	Single compression; Cut; Three-point bend	Similar	Cryo-SEM	
		100 °C, 20 min	Increased hardness over 14 days; Peak cutting force was significantly higher	Single compression; Cut	Less damage, smaller gaps in the cells, and less cell separation	Cryo-SEM	
	600 MPa, 5 min, 25 °C	Untreated	Hardness, crunchiness index, and force-deformation curve slope decreased; Max shear force increased	Puncture, Compression, Shear	n.d.	n.d.	[72]
		105 °C, 5 min	Hardness, crunchiness index, and force-deformation curve slope were greater	Puncture, Compression, Shear	n.d.	n.d.	
		85 °C, 23 min	Same hardness, crunchiness index, and force-deformation curve slope	Puncture, Compression, Shear	n.d.	n.d.	
	Pre-treatment: 600 MPa, 5 min, 25 °C Treatment: 105 °C, 5 min	Untreated & 85 °C, 23 min	Hardness, crunchiness index, and force-deformation curve slope decreased	Puncture, Compression, Shear	n.d.	n.d.	
		105 °C, 5 min	Hardness, crunchiness index, and force-deformation curve slope were greater	Puncture, Compression, Shear	n.d.	n.d.	
	Pre-treatment: 85 °C, 23 min Treatment: Stepwise compression to 600 MPa to match the thermal history of 105 °C, 5 min						
		Untreated	Hardness, crunchiness index, and force-deformation curve slope decreased	Puncture, Compression, Shear	n.d.	n.d.	
		85 °C, 23 min	Hardness and crunchiness index decreased; Force-deformation curve slope was the same	Puncture, Compression, Shear	n.d.	n.d.	
Celery	100–400 MPa, 5–60 min	Untreated	Initial pulse softening increased with pressure; Texture loss was most pronounced >200 MPa; Firmness increased with holding time	Single compression	n.d.	n.d.	[55]
Cocoyam	600 MPa, 5 and 30 min, 8–29 °C	Untreated	Maximum cutting force decreased and had a negative relationship with holding time	Cut	600 MPa for 5 min had undefined cells	LM	[57]
Green bean	500 MPa, 1 min	Untreated	Retained up to 60% firmness of control at day 0 and higher than control after 31 days	Cut	n.d.	n.d.	[58]
		Pre-treatments: 75–90 °C, 2–4 min; Treatments: Canned (118 °C, 30 min); Pulsed-HPP (1000 MPa, 80 s, 75 °C; 30 s rest; 1000 MPa); Forced air blanched (10 min) and freeze-thawed	Significantly firmer and maintained firmness over 31 days	Cut	n.d.	n.d.	
Jicama	600 MPa, 5 min, 25 °C	Untreated	Hardness was the same; Force-deformation curve slope and crunchiness index decreased	Puncture, Compression, Shear	n.d.	n.d.	[72]
		105 °C, 5 min	Hardness, crunchiness index, and force-deformation curve slope were greater	Puncture, Compression, Shear	n.d.	n.d.	
		85 °C, 23 min	Hardness increased; Force-deformation curve slope decreased; Crunchiness index was the same	Puncture, Compression, Shear	n.d.	n.d.	
	Pre-treatment: 600 MPa, 5 min, 25 °C Treatment: 105 °C, 5 min	Untreated	Hardness, crunchiness index, and force-deformation curve slope decreased	Puncture, Compression, Shear	n.d.	n.d.	
		105 °C, 5 min	Hardness, crunchiness index, and force-deformation curve slope were greater	Puncture, Compression, Shear	n.d.	n.d.	
		85 °C, 23 min	No change in hardness, force-deformation curve slope, and crunchiness index	Puncture, Compression, Shear	n.d.	n.d.	
	Pre-treatment: 85 °C, 23 min Treatment: 600 MPa, 105 °C, 5 min	Untreated	Hardness, crunchiness index, and force-deformation curve slope decreased	Puncture, Compression, Shear	n.d.	n.d.	
		105 °C, 5 min	Hardness, crunchiness index, and force-deformation curve slope were greater	Puncture, Compression, Shear	n.d.	n.d.	
		85 °C, 23 min	Hardness and crunchiness index were the same; Force-deformation curve slope was lower	Puncture, Compression, Shear	n.d.	n.d.	
	Pre-treatment: 85 °C, 23 min Treatment: Stepwise compression to 600 MPa to match the thermal history of 105 °C, 5 min	Untreated	Hardness was the same; Force-deformation curve slope and crunchiness index decreased	Puncture, Compression, Shear	n.d.	n.d.	
		105 °C, 5 min	Hardness, crunchiness index, and force-deformation curve slope were greater	Puncture, Compression, Shear	n.d.	n.d.	
		85 °C, 23 min	Hardness increased; No change in force-deformation curve slope and crunchiness index	Puncture, Compression, Shear	n.d.	n.d.	
Kohlrabi (Pickled)	400 MPa, 10–30 min	Untreated	Hardness, springiness, and chewiness decreased and had a negative relationship with holding time	Double compression	Similar cell size, shape, and intercellular spaces as control, but the middle lamella was scattered and loose	Transmission electron microscope (TEM)	[44]
		90 °C, 10–30 min	Increased hardness and springiness; No difference in chewiness	Double compression	Smaller intercellular spaces and more regular cell shapes		
Mango	300–600 MPa, 5 min, ≤30 °C	Untreated	Increates hardness; Adding calcium increased hardness	Puncture	n.d.	n.d.	[63]
Olive	400 and 600 MPa, 5 and 10 min, 14 °C	Untreated	All HPP samples were insignificantly different and maintained firmness for 186 days; Increasing storage temperature (from 15–22 °C to 30 °C) led to greater softening for all samples	Cut	n.d.	n.d.	[73]
Onion	50–600 MPa, 5 min, 20–35 °C, Vacuum packed	Untreated, With and without vacuum packing	Tissue stiffness decreased >200 MPa	Puncture	50 MPa had similar cell viability; Air spaces decreased with increasing pressure and no air or viable cells were present ≥300 MPa	LM	[74]
		40–90 °C, 30 min	300–600 MPa had similar visoelastic initial response to control at 60–90 °C but less than that at 40–50 °C	Puncture	No viable cells or air spaces were present ≥60 °C and ≥300 MPa	LM	
		40–90 °C, 30 min, Vacuum packed	Stiffness was similar at 50 MPa and ≤50 °C, and decreased ≥60 °C and ≥300 MPa, but pressure treatments decreased more	Puncture	n.d.	n.d.	[52]
Orange	100 and 200 MPa, 15–60 min	Untreated	Initial pulse softening increased with pressure; Firmness increased with holding time	Single compression	n.d.	n.d.	[55]
Peach	600 MPa, 5–30 min	90 °C, 20 min	Hardness was greater and maintained better with increased holding time, but decreased over shelf-life	Double compression	Structure was similar but with less damage; Extracellular spaces decreased as holding time increased	LM	[46]
	500 MPa, 5 min, 20 °C, Vacuum packed	Untreated	Decreased hardness and chewiness at day 0, but higher at day 21; Increased cohesiveness at day 14 and beyond; No difference in springiness	Double compression	n.d.	n.d.	[60]
		Untreated, Vacuum packed	Insignificantly different hardness, cohesiveness, and springiness; Increased chewiness at day 21	Double compression	n.d.	n.d.	
	600 MPa, 5 min, 22–38 °C	Untreated	n.d.	n.d.	Irregular cells with smaller intercellular spaces and little or no cytoplasmic material; Cell wall hydrated, swelled, and unfolded	LM; TEM	[47]
	400–600 MPa, 1–9 min, 21–38 °C		Increased pressure was associated with a greater deformation distance but did not effect other parameters; Hardness and chewiness decreased as holding time increased	Double compression	Greater cell membrane lysis; Holding time > 5 min experienced cell wall breakdown	LM	[53]
Pear	100 and 200 MPa, 15–60 min	Untreated	Initial pulse softening increased with pressure; Firmness increased with holding time	Single compression	n.d.	n.d.	[55]
Pepper (Fermented)	500 MPa, 5 min, 20 and 50 °C	Untreated	Firmness decreased but better maintained over 12 weeks	Single compression	n.d.	n.d.	[75]
		83 °C, 15 min	Increased firmness and maintained better over 12 weeks	Single compression	n.d.	n.d.	
	500 MPa, 5 min, 50 °C	80 °C, 15 min	Significantly harder; Hardness increased during storage, then decreased, whereas control sample’s hardness decreased		Water-soluble pectin was more linear; Chelate-soluble pectin fractions were similarly long branched, but more stable over 30 days; Sodium-carbonate-soluble pectin fractions were smaller, but grew over time	Atomic force microscope	[76]
Pepper (Green and Red)	100–400 MPa, 5–60 min	Untreated	Initial pulse softening increased with pressure; Texture loss was most pronounced >200 MPa; Increased holding time increased firmness for all pressures (red pepper) or only at 100 MPa (green pepper)	Single compression	n.d.	n.d.	[55]
	100 and 200 MPa, 10 and 20 min, 18–26 °C		Insignificant difference from control	Puncture	n.d.	n.d.	[35]
		70–98 °C, 1 and 2.5 min	No difference in flesh firmness; Green pepper had firmer skin than the most intense blanching treatment; Red pepper skin was firmer than all treatments, except 98 °C, 1 min, which was the same	Puncture	n.d.	n.d.	
	100–500 MPa, 15 min, 25 °C	Untreated	Firmness, hardness, cohesiveness, chewiness, gumminess, and shear force decreased, with 500 MPa decreasing the least; Springiness was insignificantly different, except endocarp treated with 100 and 300 MPa, which increased	Double compression; Cut	Cell wall swelling and separation, cell elongating, middle lamella dissolution, and cell membrane rupture, leakage, and withdrawal, especially at higher pressures	LM; TEM	[49]
		70 °C, 10 min	Similar results as compared to untreated except no differences in springiness and cohesiveness decreased, except endocarp treated with 500 MPa, which was insignificantly different	Double compression; Cut	More parenchymal tissue breakdown; Less cell wall structure; Similar cell membrane breakdown	LM; TEM	
Persimmon (Astringent)	200 MPa, 6 min, 25 °C	Untreated	Decrease in firmness, cohesiveness, and shear force	Double compression; Cut	Cell structure compacted, deformed, and spread; Middle lamella thickened and broke; Cell-cell separation; Cell membrane remained intact; Solute leakage and tannin precipitation	LM; TEM; Cryo-SEM	[59]
		70 °C, 15 min	Decrease in firmness, cohesiveness, and shear force	Double compression; Cut	Larger cells and less deformation; Thicker cell walls; Both resulted in tannin precipitation; Less withdrawal from cell wall	LM; TEM; Cryo-SEM	
Peruvian Carrot	600 MPa, 5 and 30 min, 8–29 °C	Untreated	Maximum cutting force decreased but was not affected by holding time	Cut	Starch granules gelatinized, birefringence was lost, and Maltese crosses reduced as holding time increased	LM	[57]
Pineapple	100 and 200 MPa, 15–60 min	Untreated	Initial pulse softening increased with pressure; Firmness increased with holding time	Single compression	n.d.	n.d.	[55]
	Pre-treatment: 100–700 MPa, ≤35 °C Treatment: Same as control	Osmotic dehydration (40 °C, 50 °Bx) followed by vacuum oven drying (60 °C, 18 h)	Less force was needed to penetrate the same distance; Softening increased with pressure, but minimal additional softening > 300 MPa	Puncture	Increased cell permeability, decreased intercellular materials, water loss, and damaged cell walls; Minimal further changes > 300 MPa	LM	[51]
	600 MPa, 3 min, 4 °C	Untreated	Decreased hardness and chewiness; No difference in cohesiveness, resilience, and springiness	Double compression	Relatively unchanged, with some signs of dehydration and cell wall thickening/swelling	LM	[34]
		93.3 °C, 5 min	Increased hardness and chewiness; No difference in cohesiveness, resilience, and springiness	Double compression	n.d.	n.d.	
		Ohmic heating: ≤90 °C increase, 1900 L/h	No difference	Double compression	n.d.	n.d.	
Pumpkin	100–600 MPa, 2 min, 20–38 °C	Untreated	Hardness significantly decreased and pressures > 300 MPa were not significantly different from each other	Double compression	At 300 MPa, plasmosis, dissolving of middle lamella, and intercellular leakage; At 600 MPa, greater deformation of cell membrane and wall	TEM; LM	[41]
		100 °C, 2 min	Hardness, springiness, cohesiveness, resilience, and chewiness were all significantly higher	Double compression	n.d.	n.d.	
Red Radish	600 MPa, 5 min, 25 °C	Untreated	Increased hardness; Force-deformation curve slope and crunchiness index decreased	Puncture, Compression, Shear	n.d.	n.d.	[72]
		105 °C, 5 min	Hardness, force-deformation curve slope, and crunchiness index increased	Puncture, Compression, Shear	n.d.	n.d.	
		85 °C, 23 min	Hardness and crunchiness index increased; Force-deformation curve slope was the same	Puncture, Compression, Shear	n.d.	n.d.	
	Pre-treatment: 600 MPa, 5 min, 25 °C Treatment: 105 °C, 5 min	105 °C, 5 min					
		Untreated	Hardness, force-deformation curve slope, and crunchiness index decreased	Puncture, Compression, Shear	n.d.	n.d.	
		85 °C, 23 min					
	Pre-treatment: 85 °C, 23 min Treatment: 600 MPa, 105 °C, 5 min OR Stepwise compression to 600 MPa to match the thermal history of 105 °C, 5 min	Untreated					
		105 °C, 5 min	Hardness, force-deformation curve slope, and crunchiness index increased	Puncture, Compression, Shear	n.d.	n.d.	
		85 °C, 23 min	Hardness decreased; Increased force-deformation curve slope; Crunchiness index was the same	Puncture, Compression, Shear	n.d.	n.d.	
Strawberry	550 MPa, 10–40 min, 25 and 70 °C	Untreated	Significant loss in firmness; Infusion with PME and calcium maintained or improved firmness; Treatment time did not effect firmness at 25 °C but significantly lowered it at 70 °C	Puncture	HPPed samples at 25 °C appeared similar to control, except some irregular shapes at longer processing time (40 min); HPP at 70 °C caused more damage to the cells	LM	[61]
		70 °C, 10–40 min	HPPed samples at 25 °C had higher firmness when infused with PME and calcium compared to controls and HPPed samples at 70 °C	Puncture	HPPed samples at 25 °C had less tissue disruption; HPP at 70 °C caused more damage to the cells	LM	
Sweet Potato	600 MPa, 5 and 30 min, 8–29 °C	Untreated	Maximum cutting force decreased and cutting force had a negative relationship with holding time	Cut	Starch granules gelatinized and agglomerated, birefringence was lost, and Maltese crosses reduced as holding time increased	LM	[57]
Tomato	200–600 MPa, 20 min, 20 °C	Untreated	Softening with increased pressure ≤ 400 MPa; >400 MPa, firmness increased (but was still lower than control)	Single compression	Cell damage with large bubbles (200–300 MPa) and broad intercellular cavities (500–600 MPa); Cell rupture increased with pressure, but minimal differences > 300 MPa	LM; SEM	[22]
Zucchini	400 and 600 MPa, 1 and 5 min	Untreated	Max force decreased; Displacement distance increased; Insignificant difference in first peak force (except 400 MPa, 1 min decreased)	Puncture	Cell wall swelling and dehydration increased with holding time; Cell wall lysis at 5 min	LM	[28]
		90 °C, 2 min	Insignificantly different max force, displacement distance, and first peak force (except 400 MPa, 1 min decreased)	Puncture	n.d.	n.d.	

## References

[1] De Roeck A., Duvetter T., Fraeye I., der Plancken I.V., Sila D.N., Loey A.V., Hendrickx M. (2009). Effect of High-Pressure/High-Temperature Processing on Chemical Pectin Conversions in Relation to Fruit and Vegetable Texture. Food Chem..

[2] Noguerol A.T., Pagán M.J., García-Segovia P., Varela P. (2021). Green or Clean? Perception of Clean Label Plant-Based Products by Omnivorous, Vegan, Vegetarian and Flexitarian Consumers. Food Res. Int..

[3] Balasubramaniam V.M., Barbosa-Cánovas G.V., Lelieveld H.L.M. (2016). High Pressure Processing of Food: Principles, Technology and Applications.

[4] Tonello C., Zhang H.Q., Barbosa-Cánovas G.V., Balasubramaniam V.M., Dunne C.P., Farkas D.F., Yuan J.T.C. (2010). Case Studies on High-Pressure Processing of Foods. Nonthermal Processing Technologies for Food.

[5] Oey I., Lille M., Van Loey A., Hendrickx M. (2008). Effect of High-Pressure Processing on Colour, Texture and Flavour of Fruit- and Vegetable-Based Food Products: A Review. Trends Food Sci. Technol..

[6] Rodrigo D., Cortés C., Clynen E., Schoofs L., Loey A.V., Hendrickx M. (2006). Thermal and High-Pressure Stability of Purified Polygalacturonase and Pectinmethylesterase from Four Different Tomato Processing Varieties. Food Res. Int..

[7] Chen H., Cao S., Fang X., Mu H., Yang H., Wang X., Xu Q., Gao H. (2015). Changes in Fruit Firmness, Cell Wall Composition and Cell Wall Degrading Enzymes in Postharvest Blueberries during Storage. Sci. Hortic..

[8] Elfalleh W., Guo L., He S., Wang P., Cui J., Ma Y. (2015). Characteristics of Cell Wall Structure of Green Beans During Controlled Freezing Point Storage. Int. J. Food Prop..

[9] Terefe N.S., Buckow R., Versteeg C. (2014). Quality-Related Enzymes in Fruit and Vegetable Products: Effects of Novel Food Processing Technologies, Part 1: High-Pressure Processing. Crit. Rev. Food Sci. Nutr..

[10] Chakraborty S., Kaushik N., Rao P.S., Mishra H.N. (2014). High-Pressure Inactivation of Enzymes: A Review on Its Recent Applications on Fruit Purees and Juices. Compr. Rev. Food Sci. Food Saf..

[11] Gokul Nath K., Pandiselvam R., Sunil C.K. (2023). High-Pressure Processing: Effect on Textural Properties of Food- A Review. J. Food Eng..

[12] Peng J., Bi J., Yi J., Lyu J., Zhao Y., Xu Y., Yu Y. (2021). Characterization of Tissue-Specific Differences in Cell Wall Pectic Polysaccharides of Carrot Root. J. Food Process. Preserv..

[13] Constenla D., Lozano J. (2003). Kinetic Model of Pectin Demethylation. Lat. Am. Appl. Res..

[14] Christiaens S., Van Buggenhout S., Vandevenne E., Jolie R., Van Loey A.M., Hendrickx M.E. (2011). Towards a Better Understanding of the Pectin Structure–Function Relationship in Broccoli during Processing: Part II—Analyses with Anti-Pectin Antibodies. Food Res. Int..

[15] Brummell D.A. (2006). Cell Wall Disassembly in Ripening Fruit. Funct. Plant Biol..

[16] Diaz J.V., Anthon G.E., Barrett D.M. (2007). Nonenzymatic Degradation of Citrus Pectin and Pectate during Prolonged Heating:  Effects of pH, Temperature, and Degree of Methyl Esterification. J. Agric. Food Chem..

[17] Krall S.M., McFeeters R.F. (1998). Pectin Hydrolysis:  Effect of Temperature, Degree of Methylation, pH, and Calcium on Hydrolysis Rates. J. Agric. Food Chem..

[18] Sila D.N., Smout C., Elliot F., Loey A.V., Hendrickx M. (2006). Non-Enzymatic Depolymerization of Carrot Pectin: Toward a Better Understanding of Carrot Texture During Thermal Processing. J. Food Sci..

[19] Sila D.N., Van Buggenhout S., Duvetter T., Fraeye I., De Roeck A., Van Loey A., Hendrickx M. (2009). Pectins in Processed Fruits and Vegetables: Part II—Structure–Function Relationships. Compr. Rev. Food Sci. Food Saf..

[20] Fraeye I., Knockaert G., Buggenhout S.V., Duvetter T., Hendrickx M., Loey A.V. (2009). Enzyme Infusion and Thermal Processing of Strawberries: Pectin Conversions Related to Firmness Evolution. Food Chem..

[21] Saharan R., Sharma K.P. (2019). Production, Purification and Characterization of Pectin Lyase from *Bacillus subtilis* Isolated from Moong Beans Leaves (*Vigna radiata*). Biocatal. Agric. Biotechnol..

[22] Tangwongchai R., Ledward D.A., Ames J.M. (2000). Effect of High-Pressure Treatment on the Texture of Cherry Tomato. J. Agric. Food Chem..

[23] Rodríguez-Garayar M., Martín-Cabrejas M.A., Esteban R.M. (2017). High Hydrostatic Pressure in Astringent and Non-Astringent Persimmons to Obtain Fiber-Enriched Ingredients with Improved Functionality. Food Bioprocess Technol..

[24] Sila D.N., Smout C., Satara Y., Truong V., Loey A.V., Hendrickx M. (2007). Combined Thermal and High Pressure Effect on Carrot Pectinmethylesterase Stability and Catalytic Activity. J. Food Eng..

[25] Trejo Araya X.I., Hendrickx M., Verlinden B.E., Van Buggenhout S., Smale N.J., Stewart C., John Mawson A. (2007). Understanding Texture Changes of High Pressure Processed Fresh Carrots: A Microstructural and Biochemical Approach. J. Food Eng..

[26] Balogh T., Smout C., Nguyen B.L., Van Loey A.M., Hendrickx M.E. (2004). Thermal and High-Pressure Inactivation Kinetics of Carrot Pectinmethylesterase: From Model System to Real Foods. Innov. Food Sci. Emerg. Technol..

[27] Perera N., Gamage T.V., Wakeling L., Gamlath G.G.S., Versteeg C. (2010). Colour and Texture of Apples High Pressure Processed in Pineapple Juice. Innov. Food Sci. Emerg. Technol..

[28] Paciulli M., Ganino T., Meza I.G.M., Rinaldi M., Rodolfi M., Morbarigazzi M., Chiavaro E. (2021). High Pressure and Thermal Processing on the Quality of Zucchini Slices. Eur. Food Res. Technol..

[29] Ly-Nguyen B., Van Loey A.M., Smout C., ErenÖzcan S., Fachin D., Verlent I., Truong S.V., Duvetter T., Hendrickx M.E. (2003). Mild-Heat and High-Pressure Inactivation of Carrot Pectin Methylesterase: A Kinetic Study. J. Food Sci..

[30] Verlent I., Hendrickx M., Verbeyst L., Van Loey A. (2007). Effect of Temperature and Pressure on the Combined Action of Purified Tomato Pectinmethylesterase and Polygalacturonase in Presence of Pectin. Enzym. Microb. Technol..

[31] Ünal M.Ü., Şener A. (2015). Extraction and Characterization of Pectin Methylesterase from Alyanak Apricot (*Prunus armeniaca* L.). J. Food Sci. Technol..

[32] Balny C., Masson P. (1993). Effects of High Pressure on Proteins. Food Rev. Int..

[33] Mozhaev V.V., Heremans K., Frank J., Masson P., Balny C. (1996). High Pressure Effects on Protein Structure and Function. Proteins.

[34] Rinaldi M., Littardi P., Ganino T., Aldini A., Rodolfi M., Barbanti D., Chiavaro E. (2020). Comparison of Physical, Microstructural, Antioxidant and Enzymatic Properties of Pineapple Cubes Treated with Conventional Heating, Ohmic Heating and High-Pressure Processing. LWT.

[35] Castro S.M., Saraiva J.A., Lopes-da-Silva J.A., Delgadillo I., Loey A.V., Smout C., Hendrickx M. (2008). Effect of Thermal Blanching and of High Pressure Treatments on Sweet Green and Red Bell Pepper Fruits (*Capsicum annuum* L.). Food Chem..

[36] Sila D.N., Smout C., Vu T.S., Hendrickx M.E. (2004). Effects of High-Pressure Pretreatment and Calcium Soaking on the Texture Degradation Kinetics of Carrots during Thermal Processing. J. Food Sci..

[37] Sila D.N., Yue X., VanBuggenhout S., Smout C., Van Loey A., Hendrickx M. (2007). The Relation between (Bio-)Chemical, Morphological, and Mechanical Properties of Thermally Processed Carrots as Influenced by High-Pressure Pretreatment Condition. Eur. Food Res. Technol..

[38] Yi J., Feng H., Bi J., Zhou L., Zhou M., Cao J., Li J. (2016). High Hydrostatic Pressure Induced Physiological Changes and Physical Damages in Asparagus Spears. Postharvest Biol. Technol..

[39] Terefe N.S., Tepper P., Ullman A., Knoerzer K., Juliano P. (2016). High Pressure Thermal Processing of Pears: Effect on Endogenous Enzyme Activity and Related Quality Attributes. Innov. Food Sci. Emerg. Technol..

[40] De Roeck A., Sila D., Duvetter T., Vanloey A., Hendrickx M. (2008). Effect of High Pressure/High Temperature Processing on Cell Wall Pectic Substances in Relation to Firmness of Carrot Tissue. Food Chem..

[41] Hu X., Ma T., Ao L., Kang H., Hu X., Song Y., Liao X. (2020). Effect of High Hydrostatic Pressure Processing on Textural Properties and Microstructural Characterization of Fresh-Cut Pumpkin (*Cucurbita pepo*). J. Food Process Eng..

[42] Howard L.R., Buescher R.W. (1990). Cell Wall Characteristics and Firmness of Fresh Pack Cucumber Pickles Affected by Pasteurization and Calcium Chloride. J. Food Biochem..

[43] Wan L., Wang H., Zhu Y., Pan S., Cai R., Liu F., Pan S. (2019). Comparative Study on Gelling Properties of Low Methoxyl Pectin Prepared by High Hydrostatic Pressure-Assisted Enzymatic, Atmospheric Enzymatic, and Alkaline de-Esterification. Carbohydr. Polym..

[44] Yang Z., Duan X., Yang J., Wang H., Liu F., Xu X., Pan S. (2022). Effects of High Hydrostatic Pressure and Thermal Treatment on Texture Properties of Pickled Kohlrabi. LWT.

[45] Gosavi N.S., Polunas M., Martin D., Karwe M.V. (2021). Effect of Food Microstructure on Calcium Infusion Under High Pressure. Food Eng. Rev..

[46] Zhang F., Dong P., Feng L., Chen F., Wu J., Liao X., Hu X. (2012). Textural Changes of Yellow Peach in Pouches Processed by High Hydrostatic Pressure and Thermal Processing During Storage. Food Bioprocess Technol..

[47] Denoya G.I., Nanni M.S., Apóstolo N.M., Vaudagna S.R., Polenta G.A. (2016). Biochemical and Microstructural Assessment of Minimally Processed Peaches Subjected to High-Pressure Processing: Implications on the Freshness Condition. Innov. Food Sci. Emerg. Technol..

[48] Préstamo G., Arroyo G. (1998). High Hydrostatic Pressure Effects on Vegetable Structure. J. Food Sci..

[49] Hernández-Carrión M., Hernando I., Quiles A. (2014). High Hydrostatic Pressure Treatment as an Alternative to Pasteurization to Maintain Bioactive Compound Content and Texture in Red Sweet Pepper. Innov. Food Sci. Emerg. Technol..

[50] Préstamo G., Arroyo G. (1999). Protective Effect of Ascorbic Acid against the Browning Developed in Apple Fruit Treated with High Hydrostatic Pressure. J. Agric. Food Chem..

[51] Rastogi N.K., Niranjan K. (1998). Enhanced Mass Transfer During Osmotic Dehydration of High Pressure Treated Pineapple. J. Food Sci..

[52] Gonzalez M.E., Anthon G.E., Barrett D.M. (2010). Onion Cells After High Pressure and Thermal Processing: Comparison of Membrane Integrity Changes Using Different Analytical Methods and Impact on Tissue Texture. J. Food Sci..

[53] Denoya G.I., Polenta G.A., Apóstolo N.M., Budde C.O., Sancho A.M., Vaudagna S.R. (2016). Optimization of High Hydrostatic Pressure Processing for the Preservation of Minimally Processed Peach Pieces. Innov. Food Sci. Emerg. Technol..

[54] Trejo Araya X.I., Smale N., Zabaras D., Winley E., Forde C., Stewart C.M., Mawson A.J. (2009). Sensory Perception and Quality Attributes of High Pressure Processed Carrots in Comparison to Raw, Sous-Vide and Cooked Carrots. Innov. Food Sci. Emerg. Technol..

[55] Basak S., Ramaswamy H.S. (1998). Effect of High Pressure Processing on the Texture of Selected Fruits and Vegetables. J. Texture Stud..

[56] Cheftel J.C. (1995). Review: High-Pressure, Microbial Inactivation and Food Preservation. Food Sci. Technol. Int..

[57] de Oliveira M.M., Tribst A.A.L., de Castro Leite Júnior B.R., de Oliveira R.A., Cristianini M. (2015). Effects of High Pressure Processing on Cocoyam, Peruvian Carrot, and Sweet Potato: Changes in Microstructure, Physical Characteristics, Starch, and Drying Rate. Innov. Food Sci. Emerg. Technol..

[58] Krebbers B., Matser A.M., Koets M., Van den Berg R.W. (2002). Quality and Storage-Stability of High-Pressure Preserved Green Beans. J. Food Eng..

[59] Vázquez-Gutiérrez J.L., Hernández-Carrión M., Quiles A., Hernando I., Pérez-Munuera I. (2012). Impact of High Hydrostatic Pressures on the Structure, Diffusion of Soluble Compounds and Textural Properties of Persimmon ‘Rojo Brillante’. Food Res. Int..

[60] Denoya G.I., Vaudagna S.R., Polenta G. (2015). Effect of High Pressure Processing and Vacuum Packaging on the Preservation of Fresh-Cut Peaches. LWT Food Sci. Technol..

[61] Fraeye I., Knockaert G., Van Buggenhout S., Duvetter T., Hendrickx M., Van Loey A. (2010). Enzyme Infusion Prior to Thermal/High Pressure Processing of Strawberries: Mechanistic Insight into Firmness Evolution. Innov. Food Sci. Emerg. Technol..

[62] Gosavi N.S., Salvi D., Karwe M.V. (2019). High Pressure-Assisted Infusion of Calcium into Baby Carrots Part I: Influence of Process Variables on Calcium Infusion and Hardness of the Baby Carrots. Food Bioprocess Technol..

[63] Perdomo Lamilla C., Vaudagna S.R., Cap M., Rodriguez A. (2020). Application of High Pressure-Assisted Infusion Treatment to Mango Pieces: Effect on Quality Properties. Innov. Food Sci. Emerg. Technol..

[64] Katsaros G.J., Alexandrakis Z.S., Taoukis P.S. (2017). Kinetic Assessment of High Pressure Inactivation of Different Plant Origin Pectinmethylesterase Enzymes. Food Eng. Rev..

[65] Goodner J.K., Braddock R.J., Parish M.E. (1998). Inactivation of Pectinesterase in Orange and Grapefruit Juices by High Pressure. J. Agric. Food Chem..

[66] Duvetter T., Sila D.N., Van Buggenhout S., Jolie R., Van Loey A., Hendrickx M. (2009). Pectins in Processed Fruit and Vegetables: Part I—Stability and Catalytic Activity of Pectinases. Compr. Rev. Food Sci. Food Saf..

[67] Wicker L., Ackerley J.L., Corredig M. (2002). Clarification of Juice by Thermolabile Valencia Pectinmethylesterase Is Accelerated by Cations. J. Agric. Food Chem..

[68] Cacace F., Bottani E., Rizzi A., Vignali G. (2020). Evaluation of the Economic and Environmental Sustainability of High Pressure Processing of Foods. Innov. Food Sci. Emerg. Technol..

[69] Badwaik L.S., Gautam G., Deka S.C. (2015). Influence of Blanching on Antioxidant, Nutritional and Physical Properties of Bamboo Shoot. J. Agric. Sci. Sri Lanka.

[70] Tadapaneni R.K., Daryaei H., Krishnamurthy K., Edirisinghe I., Burton-Freeman B.M. (2014). High-Pressure Processing of Berry and Other Fruit Products: Implications for Bioactive Compounds and Food Safety. J. Agric. Food Chem..

[71] Scheidt T.B., Silva F.V.M. (2018). High Pressure Processing and Storage of Blueberries: Effect on Fruit Hardness. High Press. Res..

[72] Nguyen L.T., Tay A., Balasubramaniam V.M., Legan J.D., Turek E.J., Gupta R. (2010). Evaluating the Impact of Thermal and Pressure Treatment in Preserving Textural Quality of Selected Foods. LWT Food Sci. Technol..

[73] Pradas I., del Pino B., Peña F., Ortiz V., Moreno-Rojas J.M., Fernández-Hernández A., García-Mesa J.A. (2012). The Use of High Hydrostatic Pressure (HHP) Treatments for Table Olives Preservation. Innov. Food Sci. Emerg. Technol..

[74] Gonzalez M.E., Jernstedt J.A., Slaughter D.C., Barrett D.M. (2010). Influence of Cell Integrity on Textural Properties of Raw, High Pressure, and Thermally Processed Onions. J. Food Sci..

[75] Li J., Zhao F., Liu H., Li R., Wang Y., Liao X. (2016). Fermented Minced Pepper by High Pressure Processing, High Pressure Processing with Mild Temperature and Thermal Pasteurization. Innov. Food Sci. Emerg. Technol..

[76] Chen F., Chen Y., Wang Y., Ding S., Qin Y., Jiang L., Wang R. (2022). High Pressure Processing Improves the Texture Quality of Fermented Minced Pepper by Maintaining Pectin Characteristics during Storage. J. Food Sci..

